# Brainstem and Cerebellar Volume Loss and Associated Clinical Features in Progressive Supranuclear Palsy

**DOI:** 10.1002/acn3.70318

**Published:** 2026-01-18

**Authors:** Chloe Spiegel, Timothy P. Siejka, Cassandra Marotta, Josh J. Y. Lee, Kelly Bertram, Terence J. O'Brien, Meng Law, Lucy Vivash, Ian H. Harding

**Affiliations:** ^1^ Department of Neuroscience, School of Translational Medicine Monash University Melbourne Victoria Australia; ^2^ Department of Neurology Alfred Health Melbourne Victoria Australia; ^3^ QIMR Berghofer Medical Research Institute Brisbane Queensland Australia

**Keywords:** brainstem, cerebellum, progressive supranuclear palsy, volumetric MRI

## Abstract

**Introduction:**

Progressive Supranuclear Palsy (PSP) is a neurodegenerative ‘tauopathy’ with predominating pathology in the basal ganglia and midbrain. Caudal tau spread frequently implicates the cerebellum; however, the pattern of atrophy remains equivocal. We hypothesise that volume loss is appreciable in the cerebellum and brainstem regions—beyond the midbrain—in individuals with PSP and linked to motor and non‐motor clinical features.

**Methods:**

In this cross‐sectional observational study, volumetric brainstem and cerebellar subsegmentation of T1‐weighted magnetic resonance imaging (MRI) was performed in 37 adults with PSP. Group‐level comparisons were made with 38 adults with Parkinson's disease (PD) and 35 healthy control (HC) subjects. Regional volumes in the PSP cohort were correlated against disease severity and cognition.

**Results:**

Compared with HC, the midbrain, corpus medullare, and flocculonodular lobe were smaller in PSP (*d* = 0.90–1.2). Compared with PD, the midbrain, pons, and superior cerebellar peduncle (SCP) were smaller in PSP (*d* = 0.82–1.9). There was a positive correlation between the frontal assessment battery (FAB) and volume of the superior (*r* = 0.50) and inferior (*r* = 0.48) cerebellar posterior lobes. The PSP rating scale also correlated with SCP (*r* = −0.58) and midbrain (*r* = −0.52) volume.

**Conclusion:**

Additional regions of brainstem and cerebellar volume loss, alongside midbrain atrophy, were observed in PSP. The reported clinico‐radiologic correlations suggest a role of the cerebellum in cognitive dysfunction. These findings indicate that the cerebellum is not spared and support further work to understand the temporal course of cerebellar and cerebellar connectivity changes relative to other brain regions.

## 1 Introduction

Progressive supranuclear palsy (PSP) is a rare atypical parkinsonian disorder typically characterised by supranuclear gaze palsy, axial rigidity and postural instability. It is a ‘4‐repeat tauopathy’ with predominance of tau‐related pathology in the basal ganglia and midbrain [1]. From this epicentre, tau is postulated to spread rostrally and caudally over time, affecting numerous other regions heterogeneously across PSP variants [1]. In the most well recognised variant, PSP‐Richardson's syndrome (PSP‐RS), typical oculomotor, axial and postural features are usually seen alongside non‐motor features, in particular cognitive dysfunction. With disease progression, variable degrees of dysfunction are observed in motor and non‐motor domains. Moreover, variant presentations are increasingly recognised, often attributed to early predominant regions of neurodegeneration (beyond the midbrain). For example, frontal or parietal neurodegeneration may result in ‘cortical’ phenotypes, such as PSP‐Frontal (PSP‐F) or PSP‐Corticobasal syndrome (PSP‐CBS), whereas a striatal predominance may be associated with PSP‐parkinsonism (PSP‐P) [1, 2].

Despite increasing recognition of variable tau spread and clinical heterogeneity in PSP, neuroimaging studies have primarily focused on measures of midbrain atrophy [3]. Whilst this remains an important clinical and research focus, and can support a PSP diagnosis, understanding the profile of neurodegeneration across the whole brain is critical to disentangling symptom evolution and variability. A growing body of work using MRI and PET image analysis in PSP cohorts has described changes in other supratentorial regions, particularly frontal cortex [3]. The involvement of the cerebellum in PSP remains more equivocal, despite this structure frequently being affected by tau pathology at autopsy [1, 4]. While some MRI studies have reported significant cerebellar atrophy [5, 6, 7, 8], noting associations with cognition [9, 10], gait dysfunction [5, 10] and verbal fluency [10, 11], these are inconsistent with heterogeneous methodologies, technical limitations and small sample sizes. Many studies use whole brain voxel‐based morphometry [12], while some use manual [13] or older parcellation methods (such as the spatially unbiased infratentorial template (SUIT)) [14] to evaluate the cerebellum, which do not optimally account for the inherent complexity, individual heterogeneity and intricate anatomy in this region, and therefore may not reliably detect small volumetric changes. As a result, the specific morphology underlying these potential associations remains poorly understood. Moreover, there are conflicting reports from meta‐analyses regarding the spatial profile of cerebellar grey matter alterations in PSP [12, 15, 16, 17]. Therefore, a closer look at the infratentorial structures is warranted, particularly as cerebellar dysfunction is increasingly recognised to contribute to a broad range of clinical manifestations in diseases previously thought to spare the cerebellum [18, 19, 20].

Taken together, current evidence supports a hypothesis that variable involvement of the cerebellum and other subtentorial structures across individuals with PSP may help explain the significant variability in symptom manifestation. The aim of this study is to characterise the patterns of atrophy in the brainstem and cerebellum in PSP using MRI segmentation and morphometry approaches that are optimised for subtentorial structures, and assess clinico‐radiologic correlations with both motor and non‐motor disease features.

## 2 Methods

### 2.1 Project Design

Patients meeting the Movement Disorders Society (MDS)‐PSP diagnostic criteria were recruited prospectively from the Movement Disorders clinics at Alfred Health, Melbourne, Australia. Allocations of PSP variants were made in accordance with the MDS‐PSP diagnostic criteria by a movement disorders consultant neurologist. Sixteen PSP patients underwent clinical assessment and MRI via the Monash‐Alfred atypical parkinsonian database [21]. Twenty‐two PSP patients underwent clinical assessment and MRI via a separate study conducted at multiple Australian sites. Clinical assessments common to both pathways included the PSP rating scale (PSPRS), Hoehn and Yahr (H&Y) scale, Frontal Assessment Battery (FAB) and categorical fluency. The PSPRS is a dedicated PSP severity scale ranging from 0 (lowest severity) to 100 (greatest severity). There are six domains including historical features, mental, bulbar exam, ocular, limb and gait/midline examination. The H&Y scale ranges from 1 (unilateral parkinsonism with minimal disability) to 5 (confinement to bed or wheelchair unless aided), and the FAB comprises cognitive and behavioural tests, with scores ranging from 0 (impaired performance) to 18 (better performance). Categorical fluency is assessed over a minute, with more words per minute suggesting better performance [21].

Demographic data including age, sex and disease duration, along with MRI scans, for 39 PD and 40 healthy control (HC) comparators were retrospectively collected from the Alfred Hospital, Melbourne, Australia (ethics project number 157/19). PD diagnoses were based on the medical records. The H&Y scale was estimated for PD patients based on the clinical records available at the time of MRI.

### 2.2 Standard Protocol Approvals, Registrations and Patient Consents

Ethics approval was obtained from the Human Research Ethics Committee at the Alfred Hospital (*Monash‐Alfred atypical parkinsonian database* project number 157/19; *SEL003* project number 594/20; ACTRN12600001254987). Written informed consent was obtained from all participants recruited.

### 2.3 MRI Acquisition and Processing

All PSP patients underwent a standardised brain MRI protocol on a 3‐Tesla Siemens scanner, including a 3D T1‐weighted Magnetization Prepared Rapid Gradient Echo (MPRAGE) sequence with isotropic 0.8 mm (clinical trial) or 1.0 mm voxels (atypical parkinsonian database). Retrospectively curated MRI data for the comparator groups largely ranged in resolution from isotropic 0.9–1.25 mm voxels.

The MPRAGE images were processed to generate regional brain volume estimates using Freesurfer 7.3.2, Fastsurfer 2.2.0 and the Computational Anatomy Toolbox (CAT12) version 1113 in SPM12 version 7487 for MATLAB 9.4 (R2018a). Volumes of the midbrain, pons and medulla were calculated using the *brainstem segmentation* pipeline in Freesurfer; the estimated Total Intracranial Volume (eTIV) for each scan was also recorded. The volumes of 30 cerebellar regions were calculated using a dedicated cerebellar segmentation pipeline, *CerebNet*, in Fastsurfer [22], and then consolidated into six regions of interest (ROI): the bilateral cerebellar white matter, anterior lobe (lobules I–V), superior posterior lobe (lobules VI–VII), inferior posterior lobe (lobule VIII–IX), flocculonodular lobe (lobule X) and the vermis. ROI are shown in the Supporting Information (Figures S1 and S2).

Voxel‐based morphometry (VBM) was used to calculate volumes of the superior cerebellar peduncle (SCP), middle cerebellar peduncle (MCP), Inferior Cerebellar Peduncle (ICP) and the dentate region of the cerebellum. The T1‐weighted scans were processed in CAT12 to segment the brain into grey matter, white matter and CSF, and then nonlinearly register the white matter segment to standard MNI space. The Jacobian determinant of the registration was calculated and used to assign an estimated volume value to each voxel in MNI space, as per standard VBM approaches. The volume of each ROI was calculated by summing the values of all voxels within bilateral masks of the three peduncles provided by the John Hopkins University (JHU) atlas [23], and for the dentate nuclei using the SUIT atlas [24].

Individual differences in head size were accounted for by scaling all volume measures by eTIV prior to statistical analyses (i.e., all volumes are expressed as %eTIV). Freesurfer eTIV estimate was used for ROIs derived from the Freesurfer and Fastsurfer pipelines, while the CAT12 eTIV estimate was used for the VBM‐based ROIs.

### 2.4 Quality Control

Scans were first reviewed visually to ensure alignment. MRIQC version 24.1.0 and CAT12 were used to assess scan quality based on automated metrics of image properties including signal‐to‐noise ratio (SNR) and Full Width at Half Maximum (FWHM). Two scans were subsequently removed due to excessive image smoothness (FWHM) associated with considerable motion artefact. Using the CAT12 image and preprocessing quality metrics, one additional scan was removed due to very poor resolution (D+ 67.37%), likely due to partial volume effects.

Segmentation quality was additionally assessed by comparing the eTIV estimates derived by Freesurfer and by CAT12. Although subtle differences are expected, these estimates should be highly concordant and correlated. We noted five cases of scans with significant discrepancy (> 300 cm^3^) in these measures, suggesting a processing error in at least one of the pipelines. As outcomes from both pipelines are used in our analyses, these scans were excluded. As an additional quality check, we compared the eTIVs across sex and disease groups. As expected, the eTIV was larger in males than females (Freesurfer mean eTIV in males 1608 cm^3^ and females 1415 cm^3^; CAT12 mean eTIV in males 1627 cm^3^ and females 1433 cm^3^, *p* < 0.001). There were no significant differences across the disease groups for the CAT12 eTIV (PSP 1554 cm^3^ HC 1503 cm^3^ PD 1562^3^, *p* = 0.197); however, the Freesurfer eTIV was noted to be significantly larger in the PSP group (PSP 1588 cm^3^ HC 1475 cm^3^ PD 1501 cm^3^, *p* = 0.013). All volume measures are scaled by eTIV at the individual level to account for this potential confound in all analyses.

### 2.5 Statistical Analysis

Statistical analysis was performed using Jamovi 2.3.28. Data were reported using descriptive statistics, expressed in percentages, measures of central tendency and spread. Comparisons across multiple groups were made using one‐way ANOVA and *χ* ^2^ tests. Independent samples student's *t*‐tests were used for pairwise between‐group comparisons. Clinico‐radiologic correlations with PSPRS, FAB and Category Fluency were determined using linear regression analysis with correction for age, sex and time since diagnosis. Missing data were indicated, and analyses only included subjects with available and complete data for the variable of interest. A Bonferroni corrected *p*‐value of 0.0038 was considered significant for all analyses (raw *p*‐value threshold of 0.05 corrected for 13 regions). Outcomes meeting uncorrected *p* < 0.05 thresholds are also indicated.

## 3 Results

### 3.1 Demographics and Clinical Findings

Demographic data across PSP, PD and HC groups are reported (Table 1). All three groups comprised similar age and sex distribution. Time since diagnosis was significantly shorter in the PSP group compared with PD (2.74 years in PSP vs. 6.48 years in PD, *p* < 0.001). The median Hoehn and Yahr score was significantly higher in the PSP group compared with PD (3.5 in PSP vs. 2.0 in PD, *p* < 0.001). Clinical findings in the PSP group are reported (Table 2), with a breakdown of the variants included. This cohort included 36 individuals with probable PSP, comprising 33 PSP‐RS, 2 PSP‐CBS, 1 PSP‐P and 1 PSP‐PGF variants. The individuals with PSP‐P and PSP‐PGF had a notably longer disease duration. Statistical comparisons between variants were not performed due to the small sample sizes.

**TABLE 1:** Demographics, disease duration and severity.

	PSP (*n* = 37)	PD (*n* = 38)	HC (*n* = 35)	*p*
Age (mean (SD))	68.32 (6.65)	69.12 (11.17)	65.23 (8.37)	0.149^a^
Sex (male %)	21 (57%)	22 (58%)	18 (51%)	0.841^b^
Time since diagnosis (mean (SD))	2.74 (2.95)	6.48 (5.87)	—	< 0.001^c^
H&Y (median (range))	3.5 (3–5)	2.0 (1–4)	—	< 0.001^c^

*Note:* Statistical analysis using.

Abbreviations: H&Y, Hoehn and Yahr scale; HC, healthy controls; PD, Parkinson's disease; PSP, progressive supranuclear palsy.

^a^ One‐way ANOVA.

^b^ *χ* ^2^ test.

^c^ Student's *t*‐test.

**TABLE 2:** Clinical characteristics in PSP.

	PSP (*n* = 37)	PSP‐RS (*n* = 33)	PSP‐P (*n* = 1)	PSP‐PGF (*n* = 1)	PSP‐CBS (*n* = 2)
Time since diagnosis (years)	2.74 (2.95)	2.39 (2.47)	8.30	11.60	1.10, 1.60
PSPRS	36.8 (14.60)	36.6 (12.78)^a^	20	25	68, 68
FAB	13.3 (3.55)	13.65 (3.21)^a^	18	15	5, 8
Categorical fluency	11.6 (4.78)	12.00 (4.14)^a^	19	15	0, 4

*Note:* Results reported as Mean (SD) for groups.

Abbreviations: FAB, frontal assessment battery; PSP, progressive supranuclear palsy; PSP‐CBS, progressive supranuclear palsy‐corticobasal syndrome variant; PSP‐*P*, progressive supranuclear palsy‐Parkinsonism variant; PSP‐PGF, progressive supranuclear palsy‐progressive gait freezing variant; PSPRS, Progressive Supranuclear Palsy Rating Scale; PSP‐RS, progressive supranuclear palsy‐Richardson syndrome.

^a^ Data missing for one subject.

### 3.2 Volumetric Brainstem and Cerebellar Findings

**FIGURE 1:**
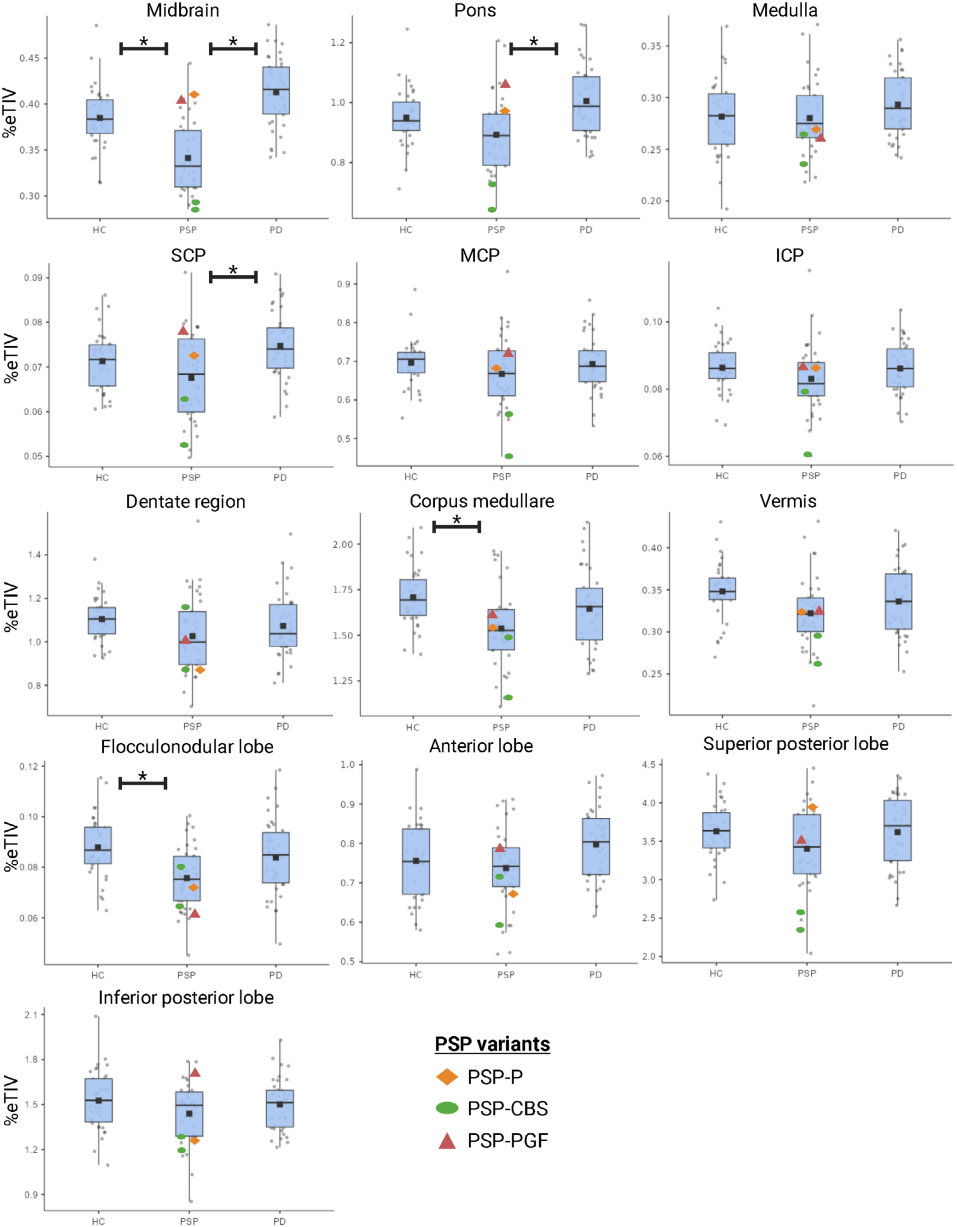
Box plots of regional volumetric comparisons with PSP variants. *p < 0.0038 (Bonferroni correction). eTIV, estimated total intracranial volume; HC, healthy controls; ICP, inferior cerebellar peduncles; MCP, middle cerebellar peduncles; PD, Parkinson's disease; PSP, progressive supranuclear palsy; SCP, superior cerebellar peduncles.

The results of comparisons using mean percentage volume of eTIV are reported (Table 3) and data are shown (Figure 1). Comparisons using unadjusted volumes are included in the Supporting Information (Table S1).

**TABLE 3:** Comparisons of regional volumetric findings.

	PSP (%)	PSP vs. PD			PSP vs. HC		
		PD (%)	*p*	Effect size	HC (%)	*p*	Effect size
**Brainstem**							
Midbrain	0.340 (0.04)	0.413 (0.37)	< 0.001**	1.864	0.385 (0.03)	< 0.001**	1.182
Pons	0.893 (0.13)	1.005 (0.12)	< 0.001**	0.924	0.950 (0.10)	0.036*	0.503
Medulla	0.280 (0.035)	0.293 (0.03)	0.098	0.387	0.282 (0.04)	0.873	0.038
**Cerebellar peduncles**							
SCP	0.068 (0.01)	0.075 (0.01)	< 0.001**	0.817	0.071 (0.01)	0.064	0.444
MCP	0.668 (0.09)	0.693 (0.07)	0.188	0.307	0.696 (0.06)	0.129	0.362
ICP	0.083 (0.01)	0.086 (0.01)	0.145	0.341	0.086 (0.01)	0.117	0.374
**Deep CBLM**							
Dentate region	1.026 (0.18)	1.073 (0.02)	0.224	0.283	1.105 (0.10)	0.024*	0.543
Corpus medullare	1.537 (0.21)	1.645 (0.22)	0.034*	0.500	1.708 (0.16)	< 0.001**	0.896
**Cerebellar grey matter**							
Anterior lobe	0.738 (0.10)	0.798 (0.09)	0.007*	0.642	0.756 (0.10)	0.425	0.189
Superior posterior lobe	3.404 (0.51)	3.620 (0.46)	0.059	0.443	3.628 (0.36)	0.037*	0.502
Inferior posterior lobe	1.440 (0.21)	1.501 (0.17)	0.169	0.321	1.526 (0.20)	0.079	0.420
Flocculonodular lobe	0.076 (0.01)	0.084 (0.02)	0.013*	0.589	0.088 (0.01)	< 0.001**	0.986
Vermis	0.322 (0.04)	0.336 (0.04)	0.150	0.336	0.348 (0.03)	0.006*	0.665

*Note:* Regional volume as percentage of eTIV reported as Mean (SD) for each group. *p* values and Effect Sizes calculated using student's *t*‐test and Cohen's *d*. Compared with HC significantly lower volumes were observed in the midbrain, corpus medullare and flocculonodular lobe. Compared with PD significantly lower volumes were observed in the midbrain, pons and SCP.

Abbreviations: CBLM, cerebellum; HC, healthy controls; ICP, inferior cerebellar peduncle; MCP, middle cerebellar peduncles; PD, Parkinson's disease; PSP, progressive supranuclear palsy; SCP, superior cerebellar peduncles.

* *p* < 0.05.

** *p* < 0.0038 (Bonferroni correction).

#### 3.2.1 PSP versus HC

Midbrain volume was significantly lower in PSP compared with HC with a very large effect size (*d* = 1.2, *p* < 0.001). A medium effect size group difference in pons volume was also observed (*d* = 0.50, *p* = 0.036), but did not survive correction for multiple comparisons. In the cerebellum, the corpus medullare (*d* = 0.90, *p* < 0.001) and flocculonodular lobe (*d* = 0.99, *p* < 0.001) were significantly reduced compared with HC with large effect sizes. Trends towards reduced volume with medium effect sizes were also observed in the dentate region (*d* = 0.54, *p* = 0.024), vermis (*d* = 0.67, *p* = 0.006) and superior posterior lobe (*d* = 0.50, *p* = 0.037); however, these did not survive correction for multiple comparisons.

#### 3.2.2 PSP versus PD

Compared with PD, midbrain (*d* = 1.9, *p* < 0.001), pons (*d* = 0.92, *p* < 0.001) and SCP (*d* = 0.82, *p* < 0.001) volumes were significantly lower in PSP. Medium effect size differences in the anterior lobe (*d* = 0.64, *p* = 0.007), cerebellar white matter (*d* = 0.50, *p* = 0.034) and flocculonodular lobe (*d* = 0.59, *p* = 0.013) were also observed, but did not survive correction. Analysis of the PSP‐RS group alone, omitting the other rare variants, versus HC and PD revealed the same pattern of results with only minor deviations in effect size and significance estimates.

### 3.3 Clinico‐Radiologic Correlations

Findings for each clinical variable are reported for the entire PSP group (Table 4) and for the PSP‐RS only group (Table 5). Scatterplots are shown for select regions where significant correlations were observed with the PSPRS and FAB (Figure S3).

**TABLE 4:** Clinico‐radiologic correlations (including all variants).

		Midbrain	Pons	Medulla	SCP	MCP	ICP	Dentate region	Corpus medullare	Vermis	Flocculo‐nodular lobe	Anterior lobe	Superior posterior lobe	Inferior posterior lobe
Years since diagnosis	Pearson's *r*	0.118	0.149	−0.283	0.040	−0.045	−0.108	−0.056	−0.043	0.074	−0.167	−0.030	0.035	0.071
(*n* = 37)	*p*	0.486	0.377	0.090	0.813	0.792	0.524	0.741	0.803	0.663	0.323	0.860	0.835	0.675
PSPRS	Pearson's *r*	−0.529	−0.328	−0.408	−0.583	−0.405	−0.468	0.313	−0.217	0.071	−0.001	0.030	−0.164	−0.070
(*n* = 36)	*p*	0.002	0.062	0.018	< 0.001	0.020	0.006	0.076	0.225	0.696	0.996	0.867	0.363	0.700
FAB	Pearson's *r*	0.170	0.236	0.093	0.130	0.285	0.238	−0.082	0.322	0.398	0.081	0.277	0.502	0.494
(*n* = 36)	*p*	0.344	0.186	0.607	0.471	0.108	0.183	0.650	0.068	0.022	0.656	0.118	0.003	0.003
Categorical Fluency	Pearson's *r*	0.340	0.225	0.138	0.282	0.323	0.287	0.028	0.155	0.252	−0.110	−0.062	0.359	0.275
(*n* = 36)	*p*	0.053	0.208	0.445	0.112	0.067	0.105	0.876	0.390	0.158	0.541	0.730	0.040	0.121

*Note:* Blue = positive correlation, Orange = negative correlation, lighter = *p* < 0.05 (significance at uncorrected threshold), darker = *p* < 0.0038 (significance at Bonferroni corrected threshold). Worse disease severity significantly correlates with lower midbrain and SCP volumes, while worse cognitive impairment significantly correlates with lower superior and inferior posterior lobe volumes.

Abbreviations: eTIV, estimated total intracranial volume; FAB, frontal assessment battery; HC, healthy controls; ICP, inferior cerebellar peduncles; MCP, middle cerebellar peduncles; PD, Parkinson's disease; PSP, progressive supranuclear palsy; PSPRS, Progressive Supranuclear Palsy Rating Scale; SCP, superior cerebellar peduncles.

**TABLE 5:** Clinico‐radiologic correlations (including PSP‐RS only).

		Midbrain	Pons	Medulla	SCP	MCP	ICP	Dentate region	Corpus medullare	Vermis	Flocculo‐nodular lobe	Anterior lobe	Superior posterior lobe	Inferior posterior lobe
Years since diagnosis	Pearson's *r*	−0.168	−0.026	−0.340	−0.144	−0.199	−0.276	0.003	−0.132	0.063	−0.046	−0.060	−0.091	−0.014
(*n* = 33)	*p*	0.350	0.887	0.053	0.422	0.266	0.120	0.988	0.465	0.726	0.801	0.741	0.616	0.938
PSPRS	Pearson's *r*	−0.315	−0.080	−0.299	−0.483	−0.144	−0.253	0.362	0.007	0.262	−0.046	0.182	0.106	0.095
(*n* = 32)	*p*	0.096	0.679	0.115	0.008	0.457	0.186	0.054	0.972	0.169	0.813	0.345	0.585	0.623
FAB	Pearson's *r*	−0.069	0.083	−0.029	−0.014	0.141	0.108	−0.005	0.296	0.404	0.158	0.295	0.411	0.527
(*n* = 32)	*p*	0.724	0.667	0.883	0.945	0.467	0.576	0.979	0.119	0.030	0.414	0.120	0.027	0.003
Categorical Fluency	Pearson's *r*	0.145	0.058	0.020	0.177	0.185	0.168	0.146	0.075	0.230	−0.073	−0.127	0.219	0.255
(*n* = 32)	*p*	0.452	0.766	0.917	0.357	0.337	0.384	0.450	0.699	0.231	0.707	0.510	0.254	0.181

*Note:* Blue = positive correlation, Orange = negative correlation, lighter = *p* < 0.05 (significance at uncorrected threshold), darker = *p* < 0.0038 (significance at Bonferroni corrected threshold). Worse cognitive impairment significantly correlates with lower inferior posterior lobe volume.

Abbreviations: eTIV, estimated total intracranial volume; FAB, frontal assessment battery; HC, healthy controls; ICP, inferior cerebellar peduncles; MCP, middle cerebellar peduncles; PD, Parkinson's disease; PSP, progressive supranuclear palsy; PSPRS, Progressive Supranuclear Palsy Rating Scale; SCP, superior cerebellar peduncles.

#### 3.3.1 Disease Duration

There were no regional volumes that significantly correlated with time since diagnosis. A separate analysis with only PSP‐RS (variants excluded) also did not show any significant correlations.

#### 3.3.2 Disease Severity

The PSPRS was observed to negatively correlate at corrected‐level significance with midbrain (*p* = 0.002, *R* = −0.529) and SCP (*p* < 0.001, *R* = −0.583) volumes. Negative correlations between medulla (*p* = 0.018, *R* = −0.408), MCP (*p* = 0.020, *R* = −0.405) and ICP (*p* = 0.006, *R* = −0.468) were also present at uncorrected significance. No significant correlations were observed with cerebellar ROIs. The analysis of PSP‐RS only showed a similar negative correlation with SCP volume (*p* = 0.008, *R* = −0.483) which did not meet the corrected significance threshold. Analysis for each PSPRS sub‐score is shown in Tables S3 and S4. Higher bulbar scores significantly correlate with lower midbrain volume. Higher mentation, bulbar, and ocular motor scores significantly correlate with lower SCP volume (Table S3); however, when including only PSP‐RS, none of these correlations survived correction for multiple comparisons (Table S4).

#### 3.3.3 Cognition

There was a significant positive correlation between the FAB and cerebellar superior posterior (*p* = 0.003, *R* = 0.502) and inferior posterior (*p* = 0.003, *R* = 0.484) lobe volumes. Analysis of only PSP‐RS showed the same relationship between the FAB and posterior lobe regions (inferior: *p* = 0.003, *R* = 0.527; superior: *p* = 0.027, *R* = 0.411), but which no longer reached corrected significance in the superior posterior region. A similar trend was noted between the FAB and the vermis volume (*p* = 0.022, *R* = 0.398), and between categorical fluency and cerebellar superior posterior lobe (*p* = 0.040, *R* = 0.359) volume. While the relationship between FAB and vermis (*p* = 0.030, *R* = 0.404) volume was comparable on the analysis of PSP‐RS, no similar relationships were observed with categorical fluency.

## 4 Discussion

In this study we have identified several infratentorial regions of volume loss beyond the midbrain in PSP using a dedicated segmentation pipeline that has not previously been used in this group. Of note, significant volume loss was seen in the pons and several cerebellar regions, alongside midbrain and SCP atrophy. Significant clinico‐radiologic correlations were noted with disease severity and cognition. Importantly, disease severity negatively correlated with brainstem and peduncle volumes. Cognitive performance, measured by FAB scores, was noted to positively correlate with superior and inferior cerebellar posterior lobe volumes, representing a novel finding indicating distinct cerebellar topography with potential involvement in PSP.

As expected, significant midbrain volume loss was detected in PSP compared with both Parkinson's disease and healthy control groups, and this was correlated with disease severity. This result is consistent with prior volumetric studies in PSP patients [9, 25, 26], and concordant with the known pathophysiology of this disease. Interestingly, there was no significant correlation with disease duration, which may indicate variable tau spread and neurodegeneration. Volume loss was also noted in the pons in our study, particularly in comparison with Parkinson's disease. Despite relative sparing of the caudal brainstem regions in PSP, there have been several studies reporting significant pontine atrophy [8, 27, 28, 29], including a prior meta‐analysis [30]. Similar to many prior studies [31, 32, 33, 34], we did not find significant volumetric differences in the medulla. These findings are consistent with the central involvement of the midbrain in PSP pathology, but also suggest more subtle changes in the pons that may be clinically relevant.

In the cerebellar peduncles, we report significant volume loss in the SCP relative to PD, although not in comparison with HC, and we show that SCP volume is associated with disease severity. The SCP predominantly carries efferent fibres that emerge from cerebellar dentate nuclei and form the first leg of the ascending dentato‐thalamo‐cortical pathway. SCP volume loss has been reported across many studies in PSP [26, 27, 29, 33], and appears to coincide with midbrain atrophy [26, 35]. These disproportionate regions of atrophy are well recognised and are included in several planimetry metrics, in particular Magnetic Resonance Parkinsonian Index (MRPI) [36, 37]. It is unexpected that the SCPs were not significantly smaller in comparison with HC; however, there was an apparent trend. There were no significant volumetric differences observed in the MCP and ICP, which is compatible with relative sparing of these structures. Intriguingly, despite this, the ICP volume was found to negatively correlate with disease severity, and a relationship with a similar effect size with MCP volume was also found, although it fell outside of corrected‐level significance. The MCP predominantly carries afferent fibres to the cerebellum, forming the second leg of the cortico‐ponto‐cerebellar tract, while the ICP predominantly carries afferent fibres from the spinal cord, vestibular system and lower brainstem to the cerebellum. The potential implications of damage to these structures would be wide‐ranging, affecting motor and cognitive domains by impairing vital cerebro‐cerebellar loops. Moreover, although not observed in our study, there are prior reports of volume loss in the MCPs and ICPs in PSP [33, 36]. It is feasible that small volumetric changes in the relatively spared MCPs and ICPs contribute multifactorially to disease severity, alongside the more robust changes observed in the SCPs.

In the cerebellum, significant volume loss was noted in the corpus medullare in PSP relative to healthy controls and Parkinson's disease to a lesser extent. Though reports of cerebellar changes in PSP have been inconsistent, such findings are in‐line with some prior volumetric studies [6, 7, 8, 38]. It is important to note that the corpus medullare comprises both the cerebellar white matter and deep cerebellar nuclei, including the dentate nucleus. The dentate nuclei are the primary output hubs of the cerebellum and give rise to the SCPs. Several tau PET studies have shown increased uptake in the dentate nucleus [26, 34, 39] and diffusion imaging studies have shown microstructural impairments along this pathway in PSP [8, 37]. We therefore examined the dentate region in isolation from the corpus medullare using a voxel‐based approach. While the volume of the dentate nucleus region was significantly lower in PSP compared with healthy controls, this did not survive correction and was not seen in the comparison with Parkinson's disease. This finding suggests that the volumetric difference observed in the corpus medullare is not entirely attributable to changes in the dentate nucleus, indicating the likelihood of white matter volume loss. This is consistent with reports of tau pathology in the cerebellar white matter at autopsy across multiple PSP variants [1, 4].

A consistent pattern of smaller volumes in cerebellar cortex regions was observed in the PSP cohort relative to both PD and healthy controls, but with varying magnitude across regions (*d* = 0.2–1.0) and only sparsely meets our stringent criteria for statistical significance. Whilst these observations therefore need to be interpreted with caution, and must be confirmed in a larger study cohort, this pattern suggests a relatively subtle involvement of the cerebellar cortex in PSP pathology. The clinical relevance of these observations is indicated by our finding that superior and inferior posterior lobe volumes correlated with cognitive functioning, assessed by the FAB, in the PSP cohort. Involvement of the cerebellar posterior lobe in PSP is further supported by a voxel‐based meta‐analysis from 2017 which reported significant volume loss in crus I, crus II, lobule VIIb in the superior posterior lobe, as well as lobule IX in the inferior posterior lobe in PSP [15]. Moreover, functional MRI studies have revealed connectivity of the posterior lobe with several networks underpinning cognitive function, including the executive control network, dorsal and ventral attention networks, and the default mode network [40]. Disruption of these networks is postulated to contribute to cognitive dysfunction in PSP, along with several other neurodegenerative disorders [15], however, a clear clinico‐radiologic relationship has not been established in prior studies of PSP [41]. These findings align with the current understanding of cerebellar functional topography and suggest critical involvement of the cerebellum in non‐motor PSP symptoms.

Taken together with variable reports of involvement of other cerebellar regions, including the anterior lobe and vermis, in PSP [8, 11, 12, 38], the current study provides exciting indications that emphasise the need to better characterise cerebellar alterations in PSP. Further studies of the cerebellum in association with other non‐motor symptoms in PSP, such as affect, mood and language, would be worthwhile. Clinical profiling could be improved in future studies by including additional dedicated assessments, such as a neuropsychological battery, the Unified Parkinson's Disease Rating Scale (UPDRS), modified PSPRS and specific cerebellar scales. Whilst it remains to be proven, molecular and microstructural imaging methods may add to our recognition of cerebellar involvement before the emergence of significant atrophy. Future dedicated studies of the cerebellum using these methods would potentially improve detection of significant clinico‐radiologic correlations. Lastly, longitudinal studies are needed to better understand the evolution of cerebellar changes in PSP. Improving our understanding of the cerebellum in PSP potentially contributes to our interpretation and application of in vivo imaging in clinical practice. Furthermore, in time, we may find that cerebellar changes predominate in certain variant presentations, though this requires further study.

It is also important to note that while the present findings indicate structural changes in the cerebellum, the mechanisms underlying this remain uncertain. While it is feasible that these are driven by caudal tau spread, an important consideration is that they may be driven by remote network changes, for example concomitant frontal neurodegeneration resulting from supratentorial tau spread. Further studies are needed to clarify the complete spatiotemporal patterns of atrophy in this regard. The use of tau PET and dedicated functional imaging alongside volumetry may help to improve our understanding of these changes and their relationship with PSP symptoms.

There are several strengths and limitations of our study. First, PSP is a rare condition, so our sample size of 37 is larger than in many prior studies of this disease. Additionally, we have used optimised automated MRI pipelines to objectively assess subtentorial brain regions, which enables accurate individualised estimation of regional volumes. This was the first PSP study to utilise CerebNet, a highly accurate deep‐learning pipeline to optimally quantify regional cerebellar volumes. Using specific augmentation techniques, this approach does not require preprocessing, which preserves detail, enabling rapid and accurate volumetric analysis. This approach has been shown to detect subtle cerebellar atrophy with greater sensitivity, consistently and significantly outperforming older sub‐segmentation methods [22]. We chose to limit the number of ROIs included in this study in order to limit the extent of multiple comparison corrections. However, this also limited the anatomical specificity of our inferences, including examination of smaller, lobule‐level ROIs and investigations of asymmetric changes, which could shed more light on disease morphology. An exploratory analysis could be considered in this regard; however, the interpretation would be limited due to the modest sample size. International, multisite studies that aggregate MRI data from a large number of subjects, which have been successful in this regard in other rare neurodegenerative diseases [42], may provide one way forward.

Lastly, heterogeneity within our cohort is an important limitation. While most PSP patients have probable PSP‐RS, we included individuals with several additional variants in the analysis. These are known to present differently, with varying rates and distributions of tau spread, despite core pathology in the basal ganglia and midbrain [1, 4]. Statistical comparisons between variants could not be made to further elucidate these differences due to the small number, but the variants are shown (Figure 1), and we have largely replicated the results from the main analysis when restricting the analysis only to our predominant group of patients with PSP‐RS (Table S1).

## 5 Conclusion

Alongside midbrain atrophy, this study demonstrates volume loss in additional brainstem and cerebellar regions in PSP. Most importantly, these comprise several cerebellar regions, including the flocculonodular lobe and cerebellar white matter, highlighting that the cerebellum is not entirely spared in this disease. Moreover, important clinico‐radiologic correlations were detected which suggest a potential role of the cerebellum and cerebellar connections in motor and non‐motor PSP symptoms. Most notably, cognitive dysfunction in PSP was shown to correlate with the superior and inferior posterior lobe volume, and PSP disease severity with superior cerebellar peduncle volume. These findings, alongside consistent trends indicating the potential for more widespread involvement of cerebellar cortical regions, motivate the need for further research to clarify the contribution of the cerebellum to interindividual symptom variability, and the need to incorporate the cerebellum into PSP disease models.

## Author Contributions

C.S.: research project – design, organisation and execution; analysis – design, execution and review and critique; manuscript preparation – writing of the first draft and review and critique. T.P.S.: research project – design, organisation and execution; analysis – execution. C.M.: analysis – design; manuscript preparation – review and critique. J.J.Y.L.: research project – execution; manuscript preparation – review and critique. K.B.: research project – design; manuscript preparation – review and critique. T.J.O.: research project – design; manuscript preparation – review and critique. M.L.: research project – organisation; analysis – execution. L.V.: research project – design; analysis – design and review and critique; manuscript preparation – review and critique. I.H.H.: research project – design; analysis – design and review and critique; manuscript preparation – review and critique.

## Funding

This work was supported by Australian Government, MRF1200254. C.S., T.P.S., C.M. receive an Australian Government Research Training Program Stipend. TOB's institution has received research funding and consultancy fees from UCB, Eisai, LivaNova, Supernus, Praxis Pharmaceuticals, ES Therapeutics and Biogen outside the submitted work. K.B. has received consulting fees from Abbvie, Ipsen, Encapsulate, Stada and Fight Parkinson's. L.V. reports funding from the NHMRC Medical Research Future Fund (MRF1170276, MRF1200254, MRF2023250), Multiple Sclerosis Research Association and the Department of Science Industry and Resources for unrelated projects. I.H.H. receives grants unrelated to this work from the Australian National Health and Medical Research Council and the Friedreich Ataxia Research Alliance and is a consultant for Solid Biosciences Inc.

## Conflicts of Interest

The authors declare no conflicts of interest.

## Supporting information


**Figure S1:** Brainstem and cerebellar regions of interest. Brainstem and cerebellar regions of interest in colour segments: Midbrain (orange), pons (light blue), medulla (green), anterior lobe (purple), superior posterior lobe (dark blue), vermis (red) flocculonodular lobe (yellow) and inferior posterior lobe (green), corpus medullare (white). *LEFT = sagittal view in midline CENTRE = coronal view showing cerebellar segments RIGHT = axial view at the pons level*.
**Figure S2:** The cerebellar peduncles and dentate nuclei. Cerebellar peduncle and dentate nucleus regions of interest in colour segments: SCP (yellow), MCP (red), ICP (green), dentate nucleus (blue). *LEFT = sagittal view CENTRE = coronal view segments RIGHT = axial view*. SCP = superior cerebellar peduncle, MCP = middle cerebellar peduncle, ICP = inferior cerebellar peduncle.
**Figure S3:** Scatterplots of select regions with significant clinico‐radiologic correlations. a) Midbrain and SCP volumes negatively correlates with PSPRS indicating worsening disease severity is associated with smaller volumes, b) Superior and Inferior posterior lobe volumes positively correlate with FAB scores indicating worsening cognitive impairment is associated with smaller volumes.
PSPRS = progressive supranuclear palsy rating scale, SCP = superior cerebellar peduncle, FAB = frontal assessment battery, eTIV = estimated Total Intracranial Volume.
**Table S1:** Comparisons of regional volumetric findings using unadjusted volumes.
**Table S2:** Comparisons of regional volumetric findings for PSP‐RS only.
**Table S3:** PSPRS sub‐score correlations (all variants).
**Table S4:** PSPRS sub‐score correlations (PSP‐RS only).

## Data Availability

Anonymized data from the Monash‐Alfred Atypical Parkinsonian Database are available and may be requested by a qualified investigator. Data from the ongoing SEL003 study will not be available until its completion in 2026. Data sharing requires Human Research Ethics Committee approval and a signed agreement.
