# Supplementary material for: Brainstem and Cerebellar Volume Loss and Associated Clinical Features in Progressive Supranuclear Palsy
Source: Ann Clin Transl Neurol. 2026 Jan 18;13(7):1346–56. doi: 10.1002/acn3.70318 (PMC13358556; doi:10.1002/acn3.70318)
Supplement: Supplementary file 1 — Figure S1: Brainstem and cerebellar regions of interest. Brainstem and cerebellar regions of interest in colour segments: Midbrain (orange), pons (light blue), medulla (green), anterior lobe (purple), superior posterior lobe (dark blue), vermis (red) flocculonodular lobe (yellow) and inferior posterior lobe (green), corpus medullare (white). LEFT = sagittal view in midline CENTRE = coronal view showing cerebellar segments RIGHT = axial view at the pons level. Figure S2: The cerebellar peduncles and dentate nuclei. Cerebellar peduncle and dentate nucleus regions of interest in colour segments: SCP (yellow), MCP (red), ICP (green), dentate nucleus (blue). LEFT = sagittal view CENTRE = coronal view segments RIGHT = axial view. SCP = superior cerebellar peduncle, MCP = middle cerebellar peduncle, ICP = inferior cerebellar peduncle. Figure S3: Scatterplots of select regions with significant clinico‐radiologic correlations. a) Midbrain and SCP volumes negatively correlates with PSPRS indicating worsening disease severity is associated with smaller volumes, b) Superior and Inferior posterior lobe volumes positively correlate with FAB scores indicating worsening cognitive impairment is associated with smaller volumes. PSPRS = progressive supranuclear palsy rating scale, SCP = superior cerebellar peduncle, FAB = frontal assessment battery, eTIV = estimated Total Intracranial Volume. Table S1: Comparisons of regional volumetric findings using unadjusted volumes. Table S2: Comparisons of regional volumetric findings for PSP‐RS only. Table S3: PSPRS sub‐score correlations (all variants). Table S4: PSPRS sub‐score correlations (PSP‐RS only). [file ACN3-13-1346-s001.docx]

**Supplementary Material**

**
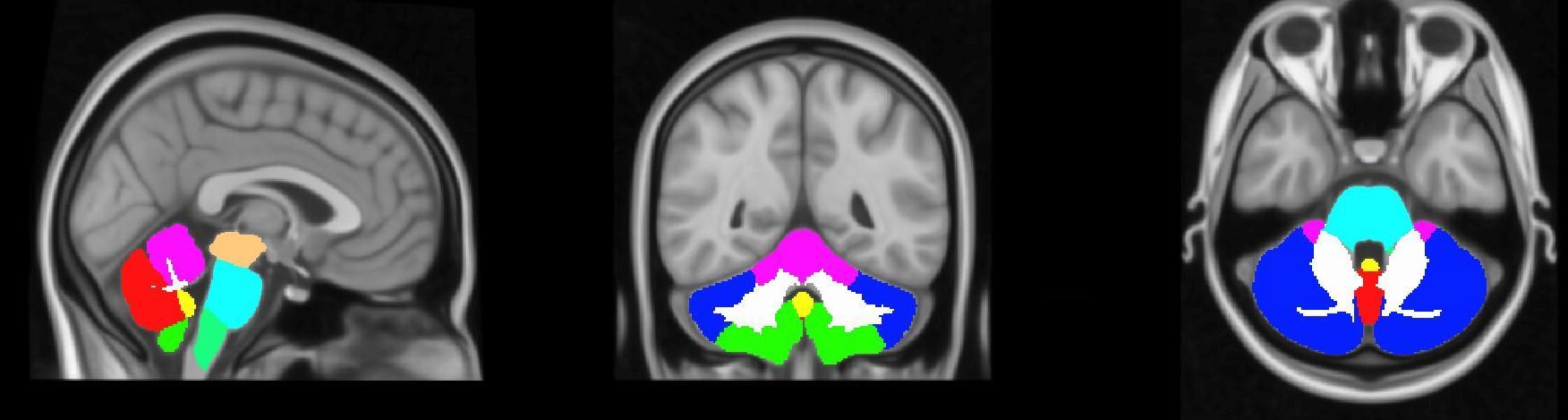
**

**Supplementary Figure 1. Brainstem and cerebellar regions of interest**

Brainstem and cerebellar regions of interest in colour segments: Midbrain (orange), pons (light blue), medulla (green), anterior lobe (purple), superior posterior lobe (dark blue), vermis (red) flocculonodular lobe (yellow) and inferior posterior lobe (green), corpus medullare (white). *LEFT = sagittal view in midline CENTRE = coronal view showing cerebellar segments RIGHT = axial view at the pons level.*


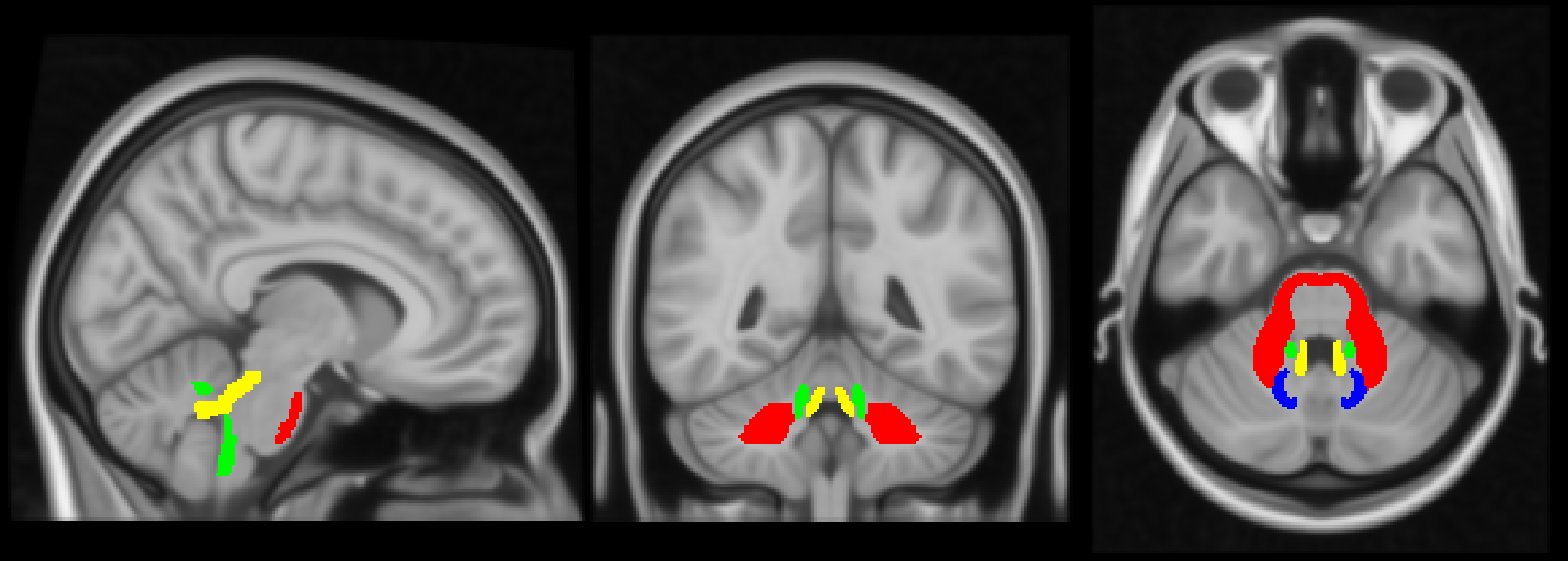


**Supplementary Figure 2. The cerebellar peduncles and dentate nuclei**

Cerebellar peduncle and dentate nucleus regions of interest in colour segments: SCP (yellow), MCP (red), ICP (green), dentate nucleus (blue). *LEFT = sagittal view CENTRE = coronal view segments RIGHT = axial view.*

SCP = superior cerebellar peduncle, MCP = middle cerebellar peduncle, ICP = inferior cerebellar peduncle


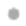


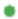


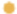


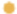


**Superior Posterior Lobe (%eTIV)**

**Inferior Posterior Lobe (%eTIV)**

**Midbrain (%eTIV)**

**SCP (%eTIV)**


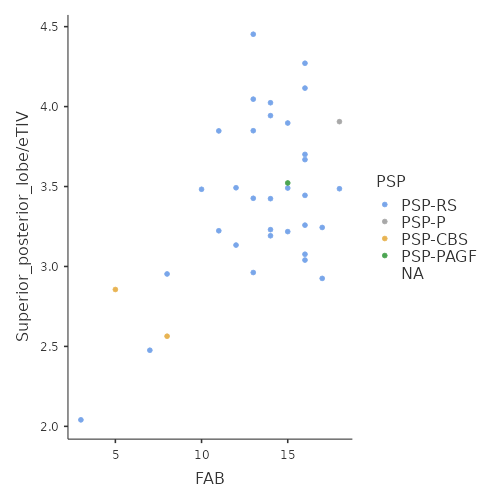


**FAB**

**FAB**


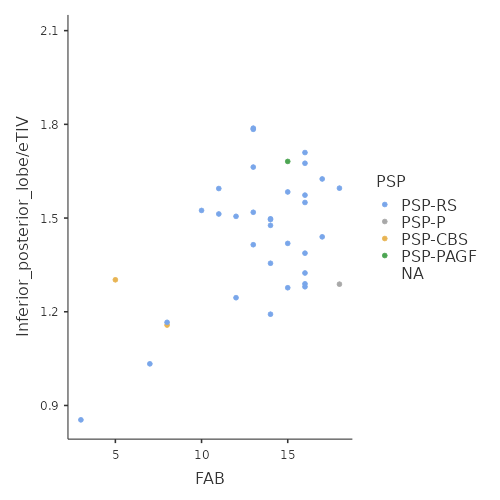

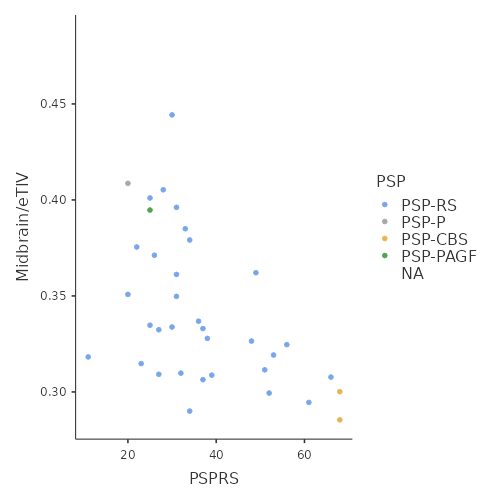


**PSPRS**


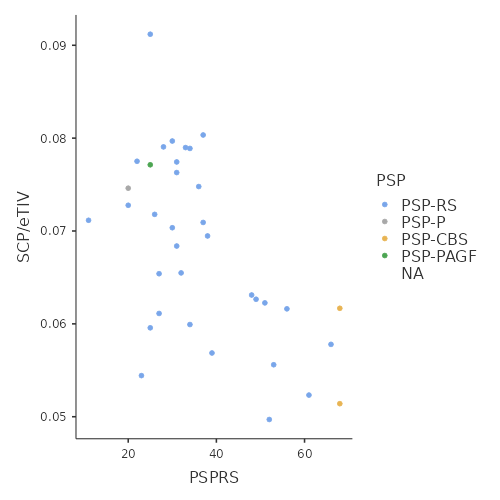


**PSPRS**

R=-0.529

p=0.002

R=-0.583

P<0.001

R=0.502

p=0.003

R=0.494

p=0.003


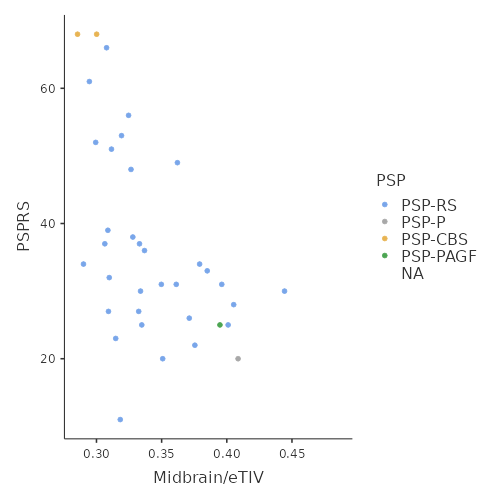


PSP-RS

PSP-P

PSP-CBS

PSP-PGF


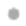


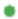


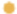


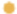


**Supplementary Figure 3. Scatterplots of select regions with significant clinico-radiologic correlations**

a) Midbrain and SCP volumes negatively correlates with PSPRS indicating worsening disease severity is associated with smaller volumes, b) Superior and Inferior posterior lobe volumes positively correlate with FAB scores indicating worsening cognitive impairment is associated with smaller volumes

PSPRS = progressive supranuclear palsy rating scale, SCP = superior cerebellar peduncle, FAB = frontal assessment battery, eTIV = estimated Total Intracranial Volume


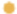

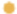


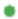


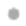


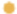

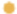


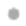

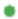


**Supplementary table 1. Comparisons of regional volumetric findings using unadjusted volumes**

|  |  | | PSP vs PD | | | PSP vs HC | | |
| --- | --- | --- | --- | --- | --- | --- | --- | --- |
|  |  | PSP (mm^3^) | PD (mm^3^) | P value | Effect Size | HC (mm^3^) | P value | Effect size |
| **Brainstem** | Midbrain | 5408 (811) | 6169 (735) | **<0.001**** | 0.985 | 5673 (663) | 0.135 | 0.357 |
|  | Pons | 14148 (2478) | 14993 (1839) | 0.097 | 0.388 | 14004 (1779) | 0.780 | -0.066 |
|  | Medulla | 4454 (792) | 4383 (586) | 0.660 | -0.102 | 4148 (608) | 0.072 | -0.431 |
| **Cerebellar Peduncles** | SCP | 1050 (182) | 1163 (152) | 0.004* | 0.677 | 1070 (116) | 0.577 | 0.132 |
|  | MCP | 10368 (1713) | 10771 (1333) | 0.258 | 0.263 | 10432 (1008) | 0.849 | 0.045 |
|  | ICP | 1290 (201) | 1341 (168) | 0.243 | 0.272 | 1295 (125) | 0.912 | 0.026 |
| **Deep CBLM** | Dentate region | 1586 (255) | 1665 (233) | 0.166 | 0.323 | 1654 (144) | 0.172 | 0.325 |
|  | Corpus Medullare | 24339 (4000) | 24578 (3656) | 0.788 | 0.062 | 25113 (2499) | 0.332 | 0.231 |
| **Cerebellar Grey Matter** | Anterior Lobe | 11665 (1766) | 11912 (1527) | 0.519 | 0.150 | 1136 (1584) | 0.186 | -0.315 |
|  | Superior Posterior Lobe | 53769 (8442) | 54072 (7829) | 0.872 | 0.037 | 53376 (5928) | 0.821 | -0.054 |
|  | Inferior Posterior Lobe | 22745 (3418) | 22466 (3310) | 0.721 | -0.083 | 22475 (3306) | 0.734 | -0.080 |
|  | Flocculonodular Lobe | 1198 (212) | 12477 (201) | 0.306 | 0.238 | 1293 (191) | 0.049* | 0.471 |
|  | Vermis | 5086 (670) | 5018 (649) | 0.660 | -0.102 | 8455 (572) | 0.804 | 0.059 |

Regional volume as percentage of eTIV reported as Mean (SD) for each group

P values and Effect Sizes calculated using student’s t- test and Cohen’s d

*p<0.05 ** p<0.0038 (Bonferroni correction)

PSP = Progressive Supranuclear Palsy, PD = Parkinson’s Disease, HC = Healthy Controls, SCP = Superior Cerebellar Peduncles, MCP = Middle Cerebellar Peduncles, ICP = Inferior Cerebellar Peduncles, CBLM = Cerebellum

**Supplementary table 2. Comparisons of regional volumetric findings for PSP-RS only**

|  |  | | PSP vs PD | | | PSP vs HC | | |
| --- | --- | --- | --- | --- | --- | --- | --- | --- |
|  |  | PSP (%) | PD (%) | P value | Effect Size | HC (%) | P value | Effect size |
| **Brainstem** | Midbrain | 0.341 (0.04) | 0.413 (0.37) | **<0.001**** | 1.944 | 0.385 (0.03) | **<0.001**** | 1.250 |
|  | Pons | 0.898 (0.12) | 1.005 (0.12) | **<0.001**** | 0.908 | 0.950 (0.10) | 0.053^a^ | 0.478 |
|  | Medulla | 0.283 (0.04) | 0.293 (0.03) | 0.200 | 0.308 | 0.282 (0.04) | 0.890 | 0.034 |
| **Cerebellar Peduncles** | SCP | 0.068 (0.01) | 0.075 (0.01) | **0.001**** | 0.808 | 0.071 (0.01) | 0.080 | 0.432 |
|  | MCP | 0.675 (0.09) | 0.693 (0.07) | 0.340 | 0.229 | 0.696 (0.06) | 0.245 | 0.285 |
|  | ICP | 0.084 (0.01) | 0.086 (0.01) | 0.233 | 0.286 | 0.086 (0.01) | 0.191 | 0.320 |
| **Deep CBLM** | Dentate region | 1.033 (0.15) | 1.073 (0.02) | 0.310 | 0.243 | 1.105 (0.10) | 0.043* | 0.500 |
|  | Corpus Medullare | 1.549 (0.21) | 1.645 (0.22) | 0.067^a^ | 0.444 | 1.708 (0.16) | **<0.001**** | 0.893 |
| **Cerebellar Grey Matter** | Anterior Lobe | 0.325 (0.04) | 0.336 (0.04) | 0.249 | 0.277 | 0.348 (0.03) | 0.016* | 0.599 |
|  | Superior Posterior Lobe | 0.077 (0.01) | 0.084 (0.02) | 0.029* | 0.529 | 0.088 (0.01) | **<0.001**** | 0.921 |
|  | Inferior Posterior Lobe | 0.733 (0.10) | 0.798 (0.09) | 0.018* | 0.579 | 0.756 (0.10) | 0.589 | 0.132 |
|  | Flocculonodular Lobe | 3.428 (0.51) | 3.620 (0.46) | 0.098 | 0.399 | 3.628 (0.36) | 0.063^a^ | 0.459 |
|  | Vermis | 1.450 (0.21) | 1.501 (0.17) | 0.260 | 0.277 | 1.526 (0.20) | 0.129 | 0.373 |

Regional volume as percentage of eTIV reported as Mean (SD) for each group

P values and Effect Sizes calculated using student’s t- test and Cohen’s d

*p<0.05 ** p<0.0038 (Bonferroni correction) ^a^Regions where p-value no longer p<0.05 with variants removedP

PSP = Progressive Supranuclear Palsy, PD = Parkinson’s Disease, HC = Healthy Controls, SCP = Superior Cerebellar Peduncles, MCP = Middle Cerebellar Peduncles, ICP = Inferior Cerebellar Peduncles, CBLM = Cerebellum

**Supplementary table 3: PSPRS sub-score correlations (all variants)**

|  | ALL PSP | Midbrain | Pons | Medulla | SCP | MCP | ICP | Dentate Region | Corpus Medull-are | Vermis | Flocculo-nodular Lobe | Anterior Lobe | Superior Post. Lobe | Inferior Post. Lobe |
| --- | --- | --- | --- | --- | --- | --- | --- | --- | --- | --- | --- | --- | --- | --- |
| **History** | Pearson's r | -0.335 | -0.147 | -0.367 | -0.401 | -0.257 | -0.321 | 0.314 | -0.127 | 0.128 | -0.15 | 0.100 | -0.014 | -0.002 |
|  | *p-value* | 0.057 | 0.413 | 0.036 | 0.021 | 0.148 | 0.068 | 0.075 | 0.480 | 0.477 | 0.404 | 0.581 | 0.938 | 0.991 |
| **Mentation** | Pearson's r | -0.490 | -0.355 | -0.328 | -0.494 | -0.409 | -0.434 | 0.245 | -0.203 | 0.039 | -0.049 | -0.036 | -0.171 | -0.140 |
|  | *p-value* | 0.004 | 0.043 | 0.063 | 0.003 | 0.018 | 0.012 | 0.169 | 0.257 | 0.827 | 0.789 | 0.844 | 0.34 | 0.438 |
| **Bulbar** | Pearson's r | -0.514 | -0.211 | -0.279 | -0.536 | -0.258 | -0.364 | 0.085 | -0.127 | 0.124 | 0.107 | 0.185 | 0.013 | 0.170 |
|  | *p-value* | 0.002 | 0.239 | 0.116 | 0.001 | 0.147 | 0.037 | 0.637 | 0.483 | 0.493 | 0.555 | 0.304 | 0.941 | 0.343 |
| **Ocular Motor** | Pearson's r | -0.361 | -0.338 | -0.279 | -0.572 | -0.463 | -0.479 | 0.179 | -0.321 | 0.039 | 0.014 | -0.046 | -0.174 | -0.126 |
|  | *p-value* | 0.039 | 0.054 | 0.116 | <0.001 | 0.007 | 0.005 | 0.318 | 0.069 | 0.829 | 0.938 | 0.798 | 0.333 | 0.483 |
| **Limb** | Pearson's r | -0.339 | -0.217 | -0.229 | -0.282 | -0.282 | -0.286 | 0.269 | -0.238 | -0.249 | -0.053 | -0.145 | -0.392 | -0.305 |
|  | *p-value* | 0.054 | 0.225 | 0.201 | 0.112 | 0.112 | 0.107 | 0.131 | 0.181 | 0.163 | 0.770 | 0.422 | 0.024 | 0.084 |
| **Gait & Midline** | Pearson's r | -0.415 | -0.193 | -0.245 | -0.376 | -0.131 | -0.247 | 0.256 | 0.003 | 0.172 | 0.107 | 0.115 | -0.012 | 0.107 |
|  | *p-value* | 0.016 | 0.281 | 0.170 | 0.031 | 0.372 | 0.166 | 0.151 | 0.986 | 0.339 | 0.552 | 0.523 | 0.948 | 0.555 |

Blue = positive correlation, Orange = negative correlation, lighter = p<0.05 (significance at uncorrected threshold), darker = p<0.0038 (significance at Bonferroni corrected threshold). Higher bulbar scores significantly correlate with lower midbrain volume. Higher mentation, bulbar and ocular motor scores significantly correlate with lower SCP volume.

**Supplementary table 4: PSPRS sub-score correlations (PSP-RS only)**

|  | PSP-RS | Midbrain | Pons | Medulla | SCP | MCP | ICP | Dentate Region | Corpus Medull-are | Vermis | Flocculo-nodular Lobe | Anterior Lobe | Superior Post. Lobe | Inferior Post. Lobe |
| --- | --- | --- | --- | --- | --- | --- | --- | --- | --- | --- | --- | --- | --- | --- |
| **History** | Pearson's r | -0.245 | -0.031 | -0.303 | -0.331 | -0.141 | -0.219 | 0.323 | -0.029 | 0.198 | -0.16 | 0.157 | 0.099 | 0.061 |
|  | *p-value* | 0.200 | 0.872 | 0.110 | 0.080 | 0.465 | 0.253 | 0.087 | 0.879 | 0.304 | 0.407 | 0.415 | 0.610 | 0.753 |
| **Mentation** | Pearson's r | -0.381 | -0.200 | -0.422 | -0.481 | -0.267 | -0.357 | 0.242 | -0.114 | 0.185 | -0.123 | 0.039 | 0.07 | -0.042 |
|  | *p-value* | 0.041 | 0.297 | 0.023 | 0.008 | 0.162 | 0.057 | 0.206 | 0.557 | 0.337 | 0.526 | 0.842 | 0.717 | 0.828 |
| **Bulbar** | Pearson's r | -0.400 | -0.135 | -0.297 | -0.497 | -0.192 | -0.308 | 0.000 | -0.063 | 0.168 | 0.084 | 0.207 | 0.148 | 0.205 |
|  | *p-value* | 0.032 | 0.484 | 0.118 | 0.006 | 0.318 | 0.104 | 1.000 | 0.744 | 0.384 | 0.667 | 0.281 | 0.445 | 0.287 |
| **Ocular Motor** | Pearson's r | -0.171 | -0.197 | -0.164 | -0.464 | -0.327 | -0.330 | 0.182 | -0.182 | 0.128 | 0.002 | 0.031 | -0.054 | -0.044 |
|  | *p-value* | 0.374 | 0.305 | 0.396 | 0.011 | 0.084 | 0.081 | 0.345 | 0.346 | 0.805 | 0.991 | 0.873 | 0.783 | 0.82 |
| **Limb** | Pearson's r | 0.000 | 0.105 | -0.036 | -0.035 | 0.058 | 0.029 | 0.294 | -0.049 | -0.201 | -0.138 | -0.082 | -0.219 | -0.266 |
|  | *p-value* | 0.999 | 0.588 | 0.854 | 0.858 | 0.765 | 0.88 | 0.121 | 0.802 | 0.296 | 0.476 | 0.672 | 0.255 | 0.164 |
| **Gait & Midline** | Pearson's r | -0.149 | 0.116 | -0.057 | -0.154 | 0.240 | 0.106 | 0.295 | 0.347 | 0.39 | 0.114 | 0.309 | 0.292 | 0.358 |
|  | *p-value* | 0.441 | 0.547 | 0.769 | 0.426 | 0.210 | 0.584 | 0.12 | 0.065 | 0.036 | 0.555 | 0.103 | 0.125 | 0.057 |

Blue = positive correlation, Orange = negative correlation, lighter = p<0.05 (significance at uncorrected threshold), darker = p<0.0038 (significance at Bonferroni corrected threshold). No significant correlations in PSP-RS only.
